# Methodological concerns with laser speckle contrast imaging in clinical evaluation of microcirculation

**DOI:** 10.1371/journal.pone.0174703

**Published:** 2017-03-30

**Authors:** Johan Zötterman, Robin Mirdell, Sandra Horsten, Simon Farnebo, Erik Tesselaar

**Affiliations:** 1 Department of Hand Surgery, Plastic Surgery and Burns and Department of Clinical and Experimental Medicine, Linköping University, Linköping, Sweden; 2 Department of Clinical and Experimental Medicine, Linköping University, Linköping, Sweden; 3 Department of Radiation Physics, Linköping University, Linköping, Sweden; University of New Mexico Health Sciences Center, UNITED STATES

## Abstract

**Background:**

Laser Speckle Contrast Imaging (LSCI) is a non-invasive and fast technique for measuring microvascular blood flow that recently has found clinical use for burn assessment and evaluation of flaps. Tissue motion caused by for example breathing or patient movements may however affect the measurements in these clinical applications, as may distance between the camera and the skin and tissue curvature. Therefore, the aims of this study were to investigate the effect of frame rate, number of frames/image, movement of the tissue, measuring distance and tissue curvature on the measured perfusion.

**Methods:**

Methyl nicotinate-induced vasodilation in the forearm skin was measured using LSCI during controlled motion at different speeds, using different combinations of frame rate and number of frames/image, and at varying camera angles and distances. Experiments were made on healthy volunteers and on a cloth soaked in a colloidal suspension of polystyrene microspheres.

**Results:**

Measured perfusion increased with tissue motion speed. The relation was independent of the absolute perfusion in the skin and of frame rate and number of frames/image. The measured perfusion decreased with increasing angles (16% at 60°, p = 0.01). Measured perfusion did not vary significantly between measurement distances from 15 to 40 cm (p = 0.77, %CV 0.9%).

**Conclusion:**

Tissue motion increases and measurement angles beyond 45° decrease the measured perfusion in LSCI. These findings have to be taken into account when LSCI is used to assess moving or curved tissue surfaces, which is common in clinical applications.

## Introduction

Laser-based techniques for measurement of skin blood flow, including Laser Doppler Flowmetry (LDF) and Laser Doppler Perfusion Imaging (LDPI), have been used during the last decades for assessment of local blood flow in tissues under stress, such as after flap surgery, burns, and poorly healing wounds[1–5]. The main drawback with LDF has been the limited measurement volume, as it is a single point measurement. The development of laser Doppler scanners (LDPI) enabled perfusion of larger tissue areas, but even with modern LDPI systems, individual scans can take up to six seconds, even though the measurement area is limited to 50–80 cm^2^[6–8].

The biomedical use of Laser Speckle Contrast Imaging (LSCI) was first introduced in the beginning of the 1980s using analog techniques. With the development of digital cameras and faster computers, it has evolved into a non-invasive, non-contact and fast technique for measuring microvascular blood perfusion. Because the technique is based on spatial variations in the speckle pattern a perfusion image can be generated in a single acquisition, which typically takes milliseconds[9].

Today, the main use of LSCI is in microvascular research, where it is used as a technique for measuring skin perfusion during tests of microvascular function. Examples of such tests are iontophoresis of vasoactive substances such as acetylcholine, post-occlusive reactive hyperemia and local heating[10, 11]. The most promising clinical application of LSCI is burn wound assessment, since it has been shown that there is a relation between burn perfusion and burn depth as well as healing time[2, 4]. The technique has recently also been used to assess the effects of partial venous occlusion on microcirculation in free flaps and on gastric tube reconstructions following esophagectomy[3, 12].

Despite the improvements from conventional laser Doppler techniques, there are still methodological concerns with LSCI that need to be addressed for the technique to gain further clinical acceptance for assessment of microcirculation[13]. A major limitation is the sensitivity of the technique to movement of the tissue that is being measured. This is particularly relevant in the assessment of burns in unsedated children and in perioperative assessment of free flaps, where artefacts due to patient movement and breathing are common, despite the short acquisition time. This may result in diagnostic errors if the technique is to be used for making clinical decisions. Another issue when LSCI is used clinically to measure larger areas is the possible effect that the measuring distance and the curvature of the tissue has on the measured perfusion. The aims of this study were therefore to investigate the effect of tissue motion on measured perfusion using different combinations of frame rate and number of frames/image and to investigate how the angle and distance between the measured surface and the LSCI camera affect the measured perfusion.

## Materials and methods

### Subjects

Ten healthy, nonsmoking subjects, mean age 32.4 (range 23–43) years, were recruited and gave their written informed consent. None of the subjects used regular medication, except for oral contraceptives. The subjects were seated comfortably in an upright position during the measurements. All provocations and measurements were done on the volar forearm after the skin had been gently cleaned with chlorhexidine ethanol (5 mg/ml, Fresenius AB, Uppsala, Sweden). All measurements were performed at a room temperature of 21 ± 1°C. The study was carried out according to the Declaration of Helsinki and was approved by the Regional Ethics Committee at Linköping University Hospital.

### Equipment

A Laser Speckle Contrast Imager (PeriCam PSI System, Perimed AB, Järfälla, Sweden) was used to measure the perfusion of the skin. The measurement principle of LSCI has previously been described in detail[9, 13]. This system uses a divergent class 1 laser with a wavelength of 785 nm to illuminate the skin. The reflected light creates a speckle pattern over the area that is illuminated. The system offers various system settings, including frame rates ranging from 1 to 92 frames per second and a selectable number of frames which can be averaged to obtain a final image. The combination of these settings affects the total image acquisition time and the noise level in the images. At a measurement distance of 25 cm, the spatial resolution of the perfusion image is 2–10 pixels/mm, dependent on the instrument settings. The system was calibrated at regular intervals as recommended by the manufacturer.

### Experimental protocol

#### Tissue motion

The effect of motion of the tissue on the measured perfusion was investigated in vitro and in vivo in the plane perpendicular to the laser beam, with the camera at 30 cm distance from the skin. A laboratory shaker (RK-30, Premiere, Shanghai, China) was used to generate a controlled, translational motion with a circular path. The shaker was set to 7 different speeds ranging from 28 mm/s to 205 mm/s (28 mm/s, 45 mm/s, 62 mm/s, 93 mm/s, 125 mm/s, 172 mm/s and 205 mm/s). The speeds were determined by calculating the circumference of the circle described by the shaker and by measuring the time it took the shaker to complete a number of 10 turns.

The effect of motion on the measured perfusion was first investigated by mounting a small dish with a cloth soaked in a colloidal suspension of polystyrene microspheres (Calibration Standard, Perimed AB, Järfälla, Sweden) on the laboratory shaker. The cloth was used to prevent the liquid from moving within the dish.

**Fig 1:**
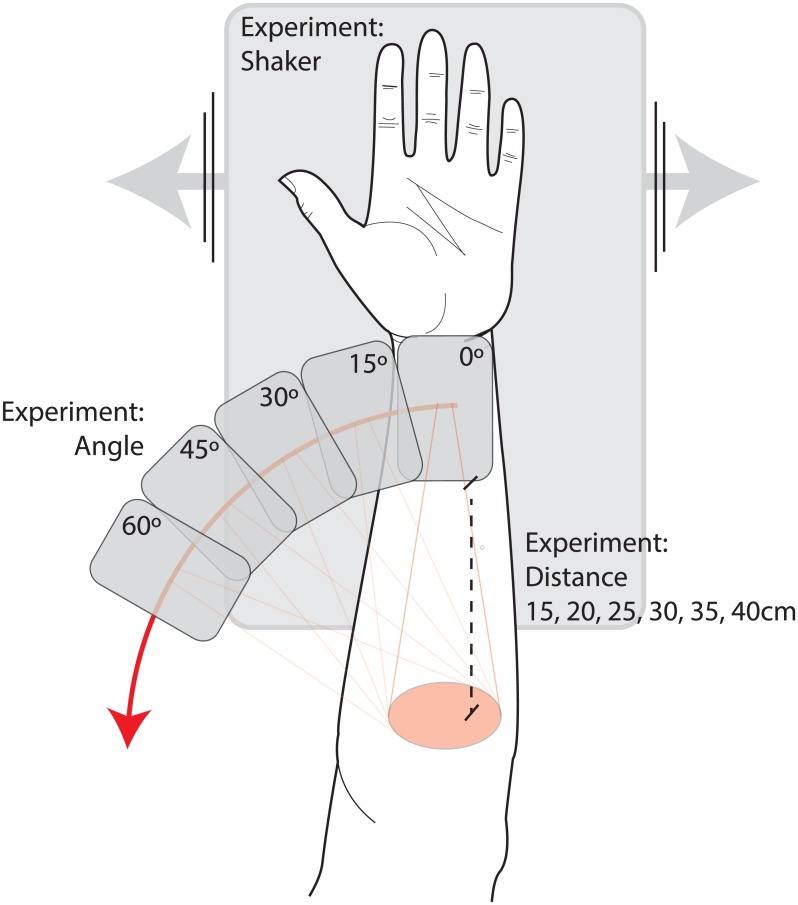
The setup of the experiment in healthy subjects. When investigating the effect of tissue motion, the forearm was resting on a laboratory shaker that generated motion at controlled speeds. When investigating the effect of camera angle and distance, the forearm was still and the camera head was tilted and moved up and down in relation to the skin surface of the forearm.

In 8 healthy subjects, the perfusion in skin of the volar side of the forearm was measured. The forearm rested on a thin pillow on the shaker platform. (Fig 1) The shaker was set to slightly lower speeds, ranging from 25 mm/s to 90 mm/s (25 mm/s, 32 mm/s, 41 mm/s, 61 mm/s and 90 mm/s). Higher speeds could not be reliably obtained because the forearm could not follow the motion at higher speed. Three circular areas with a diameter of 4 to 6 cm were marked. First, one reference measurement was made on the skin for every speed setting. Then, methyl nicotinate (MN, Sigma, St. Louis, MO, USA) was applied in three different concentrations (2.5 mM, 10 mM and 40 mM, dissolved in 70% ethanol) to the marked areas to induce a prolonged vasodilatation to simulate the higher perfusion often seen in clinical situations. Forty microliters of MN were applied on each area using a pipette and then gently spread out by hand over the marked area. When a stable vasodilatation was obtained, images were acquired using different combinations of frame rate and number of averaged frames/image, as described in Table 1.

**Table 1:** System settings used in the investigation of motion in calibration fluid and in the skin of the forearm of 8 healthy subjects.

Frame rate (s^-1^)	Number of frames per image	Measurement time (s)
21	42	2.00
10	21	2.10
5	10	2.00
21	21	1.00
21	10	0.48
21	5	0.24

The initial intention was to divide the different areas measured into groups depending on the concentration of MN applied to the skin. Because the vasodilatation was not found to be proportional to the concentration of MN, the areas were instead divided into two groups based on the measured perfusion at zero speed after application of MN. The intention of this stratification was to determine if baseline perfusion affected the impact of the speed of motion on the measurements. Since we couldn’t see any difference in perfusion between different LSCI settings we choose to use the data collected with the standard settings, that is 21 frames per second averaging 42 frames. For the first group (12 areas) the measured perfusion was lower than 135 PU. In the second group (12 areas) the perfusion was higher than 134 PU.

#### Measurement angle and distance

In 5 subjects, three of which also participated in the experiment regarding patient motion, the influence of angle and distance between the skin and the camera on measured perfusion was evaluated on the volar side of the forearm. Forty microliters of 10 mM methyl nicotinate were applied on the area using a pipette and then spread out by hand over a circular area with a diameter of 4 to 6 cm to induce a prolonged vasodilatation. When a stable vasodilatation was obtained, the arm was placed on a flat surface and the camera was then tilted to measure different angles, ranging from 0° to 60°, in steps of 15° to the plane normal to the skin at a measurement distance of 25 cm. Five images were acquired in rapid succession for each angle and averaged to obtain a mean perfusion image, using a frame rate of 21 frames/second and 42 frames/image. The effect of distance was studied in a similar way, at an angle of 0° with distances ranging between 15 and 40 cm in steps of 5 cm.

### Data analysis

All data in the text are given as mean ± SD. Images were analyzed using the LSCI system’s software (PimSoft 1.5, Perimed AB, Järfalla, Sweden). Regions of interest (ROI) were selected manually in the first image of each series. Then all subsequent images were checked to see if the selected area was still in the correct place and, if needed, corrections were made. One-way ANOVA was used to analyze the effect of measurement distance and angle. For the analysis of the effect of different speeds of motion using different absolute perfusion levels, two-way ANOVA were used. The relation between the speed of the tissue motion and the measured perfusion was analyzed using linear regression analysis. Statistical calculations were performed using GraphPad Prism version 6 for Windows (Graphpad Software, San Diego, CA, USA). A probability of less than 0.05 was accepted as significant.

## Results

### Tissue motion

**Fig 2:**
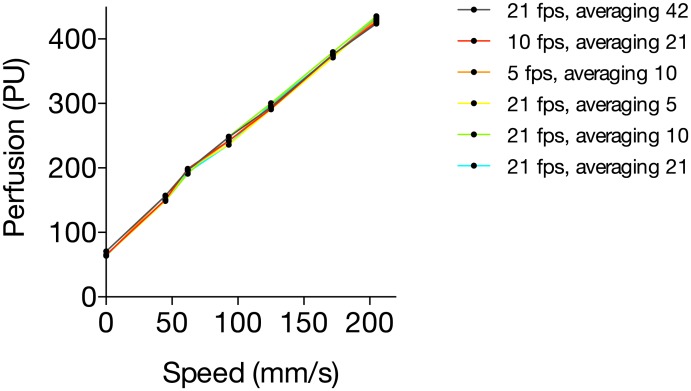
Areas in which different concentrations of methyl nicotinate were applied (1: 40 mM, 2: 10 mM, 3: 2.5 mM) on the volar side of the forearm at different speeds (A: 0 mm/s, B: 25 mm/s, C: 41 mm/s, D: 90 mm/s).

The relation between the speed of the tissue motion and the measured perfusion value in calibration fluid was linear (1.70 ± 0.02 PUmm^-1^s, linear regression analysis), varying between 66 ± 2.3 PU for zero speed and 430 ± 3.8 PU for 205 mm/s. The relation was independent of the frame rate and number of averaged image frames. The variation in perfusion between the different system settings varied between 0.7% and 3.5% (Fig 2).

**Fig 3:**
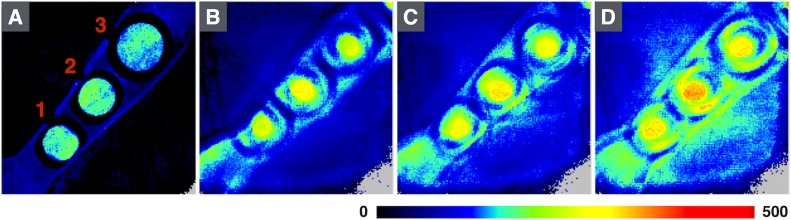
Relation between motion and measured perfusion in calibration fluid using different system settings (frame rate and number of frames).

**Fig 4:**
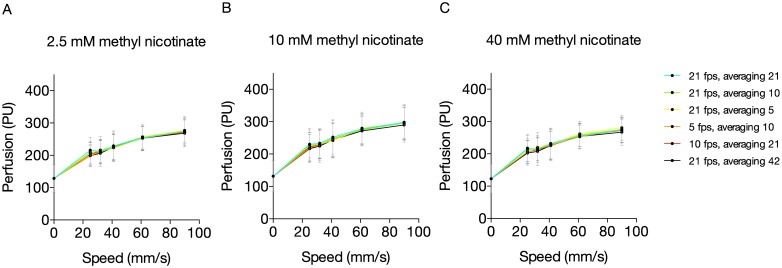
A-C. The relation between speed (mm/s) and mean measured Perfusion (PU) in the skin of the forearm after application of different concentrations of Methyl Nicotinate (MN), depending on the frame rate and the number of frames over which the perfusion is averaged (n = 8).

**Fig 5:**
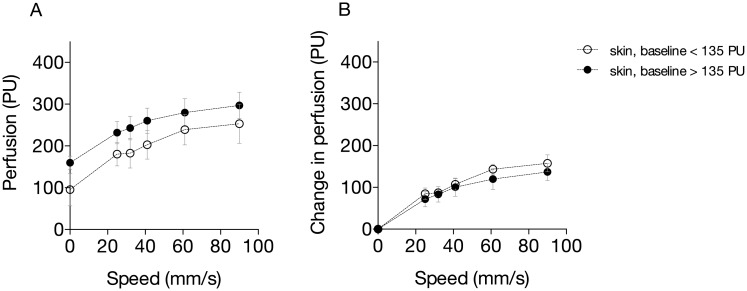
A-B. Relation between motion and measured perfusion in the skin of the forearm at different baseline perfusion values (A, absolute perfusion; and B, increase in perfusion).

After application of MN on the skin of healthy subjects, the variations in skin perfusion between different system settings were small, regardless of the speed or the concentration of MN, with coefficients of variation of less than 3%). Typical perfusion images from the measurements are shown in Fig 3. There were small but significant differences in perfusion between 2.5 mM and 10 mM and between 10 and 40 mM for all speeds (p < 0.001) (Fig 4). There were small but significant differences in the increase in perfusion in relation to motion for skin sites with different baseline perfusion levels (p = 0.03, Fig 5). Unlike the measurements in calibration fluid, the relation between the speed of the motion and the measured perfusion value in healthy subjects was not linear.

### Measurement of angle and distance

**Fig 6:**
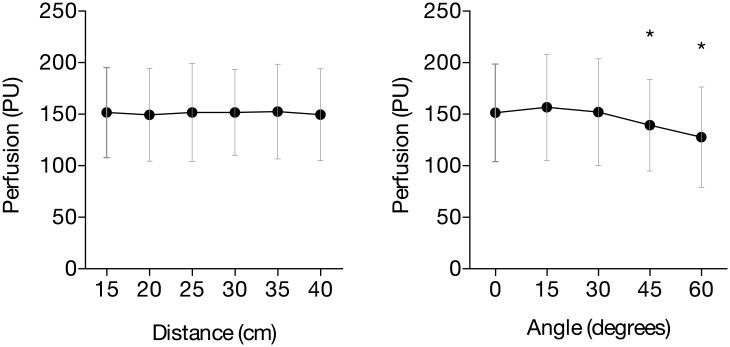
Influence of distance and angle on the measured perfusion in vivo on the dorsal side of the forearm.

The measured perfusion decreased with an increasing angle. At an angle of 45° a decrease in perfusion of 9% was observed (p = 0.03), while at 60° a decrease in perfusion of 16% was observed (p = 0.01). The decrease in perfusion when normal skin was imaged at different angles was only significant when comparing 0° to 45° (p = 0.03) and 0° to 60° (p = 0.01) but not 0° to 15° and 0° to 30°. A variation in perfusion of 0.9% was observed between different distances (p = 0.77). Both results are shown in Fig 6.

## Discussion

**Fig 7:**
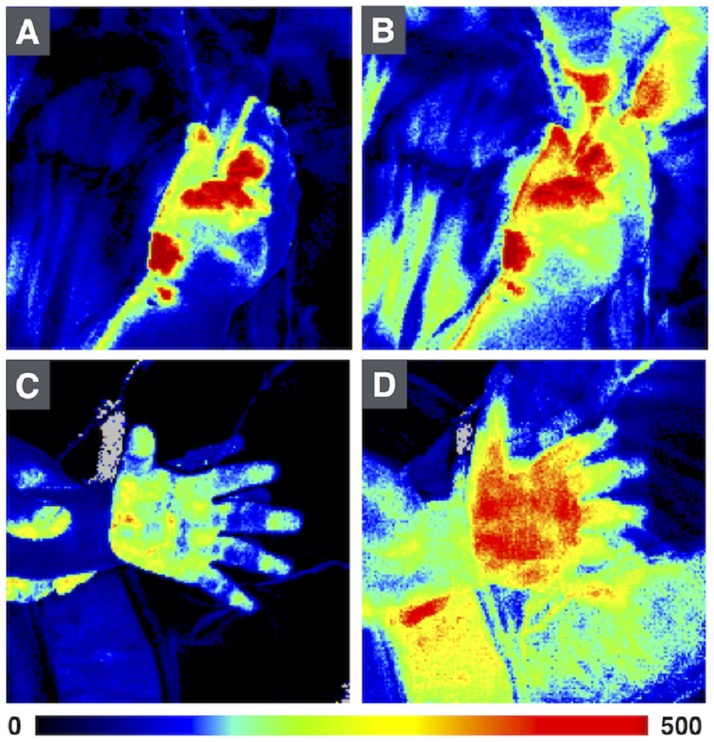
Example of images with and without motion artefacts of the hands in 2 burn patients. Images A and C do not have motion artefacts, while image B and D do. All images were taken of the same patient and injury. Note that measured perfusion is higher in images B and D, as a result of patient motion.

LSCI is camera-based technique for measuring microvascular blood flow that recently has been commercialized. Although laser Doppler imaging (LDI) is currently the only technique approved for burn assessment by the United States Food and Drug Administration (2), LSCI and LDI are actually just different ways of looking at the same phenomenon. As LSCI has the advantage that a much shorter time is needed to acquire an image, it has the potential to become a leading clinical technique for measuring tissue perfusion. The advantages in temporal resolution are especially important when measuring perfusion in young children. Still, several factors affecting the measurements have to be considered in the clinical use of the equipment, including LSCI settings, motion artefacts, curvature of the surface and distance to surface (Fig 7).

### Motion artefacts

Few studies have investigated the effect of movement artefacts on measured perfusion with LSCI. Mahé et al. tested whether an opaque adhesive patch attached close to the region of interest could be used to correct for motion artefacts using point-by-point subtraction[14]. They found a strong linear relation between the measured perfusion in the skin and in the opaque surface. The perfusion measured in the skin was 2 to 3 times higher during movement compared to the immobile situation. Although the relation between speed of motion and measured perfusion varied between subjects, this could be corrected for using the measured perfusion on the opaque surface. The optimal characteristics of the opaque adhesive patch were also evaluated[15]. The methods to produce the movement stimuli in these studies were however not standardized. Also, they used continuous measurements while we studied the effect of motion on individual images. Finally, they only performed measurements on unprovoked skin with basal perfusion.

Because we observed large variations in response to the methyl nicotinate between test persons, we grouped results based on the perfusion at zero speed to test if the absolute perfusion level had an effect on the relation between perfusion and speed. Although the measured perfusion for each speed was different between the groups, the slopes (PU/mm/s) were however similar. This suggests that the linear relation between the speed of tissue motion is fundamental to the underlying measurement principle, based on reduction of local speckle contrast, and that this is not dependent on the absolute tissue perfusion level.

When comparing the results from the measurements in the forearm of healthy subjects with those from calibration fluid, we found that the slopes were different. Although the same speeds were set on the shaking device, it is likely that the actual speed of the tissue was somewhat lower as the forearm was unable the follow the motion of the shaker at high speeds, most likely due to relative stiffness and higher weight of the forearm.

**Fig 8:**
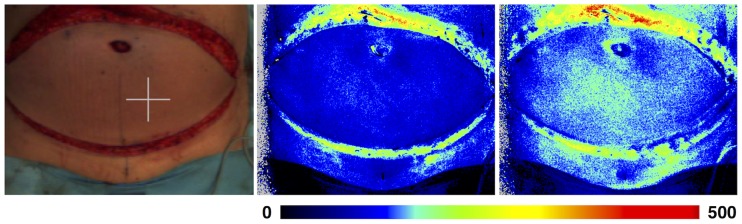
Example of breathing artifact during perfusion measurements in a Deep Inferior Epigastric Perforator (DIEP) flap. Several images were acquired during the same breathing cycle. The first image (A) is taken at end-expiratory level, when movement of the abdomen is minimal, and has a perfusion in the central region of 75 PU, whereas the second image (B) is taken at mid-expiratory level when the movement is relatively large, and a perfusion of 95 PU was measured in the same region. Thus, measured perfusion varied by 24% during the breathing cycle.

As the relation between perfusion and speed is not dependent on baseline perfusion, it might be possible to correct for patient motion if its speed could be assessed. However, this is difficult in a clinical setting. Alternatively, a reference patch could be used to correct for the effect of the motion on the measured perfusion[14]. There are some problems with this method. Because movement of patients may be present only during part of the measurement time and the speed of motion can vary, corrections would have to be made to each individual image frame, which makes data analysis laborious. Furthermore, the object of measurement will be in a different position in each frame, so all frames would have to be properly aligned. Yet another approach to mitigate the problem of motion artefacts that are of random nature, such as in burn assessment in children, is by shortening the measurement time as much as possible, as this decreases the probability that the patient moves during the image acquisition. Shorter measurement times can be obtained by lowering the number of frames/image, or by increasing the frame rate. Although the results in this study show that neither of those affect the perfusion value the noise level in the resulting perfusion image will increase as fewer frames/image are used. Thus, there are limits to how much the measurement time can be reduced while maintaining sufficient quality in the images to base clinical decisions on them. Future studies may investigate the effect of the frame rate and number of frames/image on the diagnostic quality of the perfusion images. Also, it should be noted that shortening the measurement time is not effective during constant, periodic motion such as abdominal movement due to breathing or thoracic movement due to the beating heart. In these cases, images could be acquired continuously. In this way, the images with the lowest perfusion values in the resulting series will best represent the true tissue perfusion (Fig 8).

### Angles and distances

Our results indicate that measured perfusion is not affected by the incident angle, when the camera is placed so that the laser beam points at the surface at an angle less than 45°. At 45° the decrease in PU was 9%, and at 60° the decrease was 16%.

These results agree with those in the study by Lindahl et al. in which it is stated that the measured perfusion was not dependent on incident angle for angles between 0° and 45° when measured using calibration fluid[2]. The dependency of the perfusion measurement on measurement angle seems to be weaker for LSCI than for LDI, for which a decrease in measured perfusion of 50% was found at an angle of 55°[16].

The dependency of the measured perfusion on the measurement angle has implications when measuring curved tissue, such as the extremities or abdomen, because measured perfusion in peripheral areas will be lower than in central areas with identical tissue perfusion.

We could see no significant differences in measured perfusion when assessments were obtained over distances between 15 and 40 cm. This is in accordance with the study performed by Mahé et. al in 2013, who did not find a difference in perfusion with measuring distances between 10 and 30 cm[17]. The upper limit for the camera focus is 40 cm, which is why assessment of distances beyond that would not be meaningful. Thus, we were able to verify that the measured perfusion is independent of measuring distance within the limits given by the focal length of the camera.

## Conclusion

To conclude, the findings in this study show that tissue perfusion as measured with LSCI increases with increasing tissue motion, but that this relation is independent of frame rate, number of images, and tissue perfusion. Consequently, to reduce the risk for random motion artefacts during clinical use of LSCI, image acquisition time should be kept as low as possible while maintaining adequate image quality. During periodic tissue motion, images should be acquired continuously, because in the resulting images sequence, the images with the lowest perfusion values will represent the true tissue perfusion.

The measured perfusion will decrease when images are acquired at an angle larger than 45°, and this should be considered in certain clinical settings. Distances between 15 and 40 cm do not affect the measured perfusion.

## Supporting information

S1 Supplemental Material Data from LSCI measurements. (XLSX)
